# Femoral Artery Severed and Not Ligated

**Published:** 1865-02

**Authors:** A. C. Fox


ART. IV.—*Femoral Artery Severed and not Ligated.* Reported by Assistant Surgeon A. C. FOX.

Captain A. W. Ledbetter, company "K," 9th Alabama regiment, infantry, a native of Alabama, occupation farmer, general health, prior to the war, good, was wounded near Petersburg, Va., June 29th, 1864, and admitted into hospital July 1st, 1864. Minie ball entered the left thigh at its middle third, on the inner aspect of the limb below the os femoris, ranging upward and outward, severing the femoral artery in its course, was extracted at a point about one-half an inch below the acetabulum. Upon reception of the injury there was considerable hemorrhage, which was controlled by a temporary tournequet, consisting of a handkerchief drawn tightly around the limb at its upper third. When admitted no pulsation could be detected below the wound. Temperature of the wound natural, except in the toes. Pulse 120; appetite good; cheerful and free from pain. The limb having been encased in cotton and placed in an easy position, cold water dressing was applied to the wound and absolute rest enjoined, at the same time half grain of sulphate morphine was administered to promote sleep.

July 2d.—Rested well during the night; pulse less frequent; skin moist, and no unpleasant symptoms.

The patient was put upon good diet and continued doing well. On the 14th July, pulsation was discovered in both the popliteal and tibial arteries. No unpleasant symptoms supervening, the patient was furloughed on the 30th July, 1864.

